# Combination therapy with budesonide and N-acetylcysteine ameliorates LPS-induced ALI by attenuating neutrophil recruitment through the miR-196b-5p/Socs3 molecular axis

**DOI:** 10.1186/s12890-022-02185-7

**Published:** 2022-10-26

**Authors:** Yang Li, Huimin Yu, Meifen Lv, Qiaofen Li, Kaiwen Zou, Shaokun Lv

**Affiliations:** Department of Rehabilitation Medicine, Qujing No.1 Hospital, Qujing, 655000 Yunnan China

**Keywords:** Acute lung injury, Budesonide, N-acetylcysteine, Neutrophil recruitment

## Abstract

**Background:**

Neutrophil infiltration accelerates the inflammatory response and is highly correlated to the development of acute lung injury (ALI). Budesonide (BUD) and N-acetylcysteine (NAC) both inhibit the inflammatory response to alleviate ALI, so we further investigated whether their combination is better for ALI.

**Methods:**

In this study, we investigated the effect and mechanism of Combined BUD and NAC therapy on LPS-induced ALI. Rat ALI model and neutrophil abnormal activation model were established by lipopolysaccharide (LPS). BUD and NAC were treated alone or in combination, or cells were transfected with miR-196b-5p mimic or si-Socs3 to evaluate the efficacy and mechanism of BUD and NAC alone or in combination. Histopathological observation of lungs was performed by Hematoxylin Eosin (HE) staining. The quantity of neutrophils and inflammatory factors level in bronchoalveolar lavage fluid (BALF) were determined by Richter-Gimza complex stain and Enzyme-Linked Immunosorbnent Assay (ELISA), respectively. ReverseTranscription-PolymeraseChainReaction (RT–qPCR) was utilized to assess miR-196b-5p and inflammatory factor mRNA levels. The expression level of Socs3 was detected by immunohistochemistry or Western Blot.

**Results:**

BUD and NAC combined treatment had a better effect on neutrophil recruitment and inflammatory response in LPS-induced ALI than did BUD and NAC alone. Transfection of the miR-196b-5p mimic reversed the effect of combined BUD and NAC. In conclusion, the combination of BUD and NAC is a better treatment for ALI.

**Conclusions:**

Combination therapy with BUD and NAC ameliorates LPS-induced ALI by attenuating neutrophil recruitment through the miR-196b-5p/Socs3 molecular axis.

**Supplementary Information:**

The online version contains supplementary material available at 10.1186/s12890-022-02185-7.

## Preface

Acute lung injury (ALI) is usually caused by an inflammatory response in the lungs and is related to high morbidity and mortality [1, 2]. ALI is characterized by alveolar barrier dysfunction, edema and the accumulation of immunoreactive cells in the lung [3]. Excessive activation of neutrophils is an important cause of inflammatory damage. The development of ALI is associated with massive intra-alveolar recruitment of neutrophils and lung inflammation [4]. During the pathogenesis of ALI, various inflammatory factors in the lung and circulatory system stimulate neutrophils to aggregate in the pulmonary microvascular endothelium, then adhere to the endothelium, and are activated to release a series of damage mediators, resulting in diffuse alveolar damage and eventually lead to ALI [3]. Thus, inhibiting the activation and recruitment of lung neutrophils is a possible way to alleviate inflammation in the lung.

Budesonide (BUD) and N-acetylcysteine (NAC) are often used in the treatment of bronchopneumonia [5, 6]. Researches indicated that BUD and NAC have good anti-inflammatory effects [7, 8], and Jansson AH et al. [9] found that BUD and NAC could alleviate LPS-induced ALI in rats by repressing the inflammatory response, and since both BUD and NAC are protective against ALI, we considered whether the combination of BUD and NAC would exhibit better therapeutic effects.

It has been shown that BUD and NAC regulate the expression levels of multiple microRNAs (miRNAs) during the treatment of diseases [10, 11]. At the same time, it has been reported that miRNA is involved in inflammatory response and nearly related to the pathogenesis of ALI [12]. It is reported that miR-196b-5p is associated with the progression of lung cancer [13–15], and it has been proved that miR-196b-5p plays a key role in gene regulation in lung, other respiratory and digestive tract tissues [16], but whether BUD and NAC regulate miR-196b-5p in ALI has not been reported.

Suppressor of cytokine signaling pathway 3 (Socs3) is an inflammatory inactive regulator and a key suppressor of granulopoiesis, and studies have shown that enhanced neutrophil activation-related signaling pathways are associated with Socs3 deficiency, which has a great probability to be the target of lung inflammation owing to its role of anti-inflammatory in cytokine and growth factor signaling [17]. Therefore, Socs3 could be a key factor in the treatment of inflammatory response-induced ALI. Notably, miR-196b-5p has been found to regulate Socs3 expression [18], and it is thus hypothesized that the role of Socs3 in regulating mesangial cells may be mediated by miR-196b-5p.

Based on previous findings, we speculate that BUD and NAC may inhibit the inflammatory response caused by neutrophil activation recruitment and thus alleviate LPS-induced ALI by inhibiting miR-196b-5p and upregulating Socs3 expression. In this study, we described the combined treatment of BUD and NAC in ALI rats and its potential mechanism through miR-196b-5p/Socs3 molecular axis.

## Materials and methods

### Laboratory animals

In this study, 84 SPF grade 6–8-week-old, 180–200 g male SD rats (produced from Hunan Slaughter Jingda Laboratory Animal Co., Ltd.) were picked out for the experiment. The rats were randomly assigned to the control group (NC, *n* = 12), model group (ALI, *n* = 12), budesonide treatment group (LPS + BUD, *n* = 12), N-acetylcysteine treatment group (LPS + NAC, *n* = 12) and mixed budesonide and N-acetylcysteine treatment group (LPS + co-treatment, *n* = 12). According to previous studies [19], LPS (*Escherichia coli*, 10 mg, Lot.NO.429 J032, Solarbio, China) was used for modeling: after anesthesia with sodium pentobarbital (50 mg/kg), the rats were given an endotracheal drip of LPS (5 mg/kg, dissolved in PBS), while 100 μL PBS was given to the control group. After four hours of LPS treatment, the rats were treated with 0.12, 0.24, 0.36 mg/kg BUD (2 ml: 1 mg*5 sticks, AstraZeneca Pty Ltd., Australia) or 10, 30, 60 mg/kg NAC(3 ml: 0.3 g*5 sticks, ZAMBON S.p. A, Italy), respectively, to select the best therapeutic concentration. Six mice were used at concentrations of 0.12, 0.24 mg / kg BUD or 10, 30 mg / kg NAC, respectively. Next, the optimal therapeutic concentrations of BUD and NAC (0.36 mg/kg and 60 mg/kg, respectively) were combined to treat the rats with inhalation (NAC inhalation immediately after completion of inhaled BUD). Intratracheal administration was performed with a PennCentury sprayer (Shanghai Yuyan Scientific Instrument Co., Ltd., China).Twenty-four hours later, the experiment was finished, the animals were euthanized with a high dose of 15% potassium chloride (3 mL) under deep anesthesia, and bronchoalveolar lavage fluid (BALF) was collected from the lung tissue for testing. All experimental rat protocols were approved by the Animal Ethics Committee of Kunming Medical University, and all methods were carried out following relevant guidelines and regulations. This study was carried out in compliance with the ARRIVE guidelines.

### Histological research

Rats without BALF collection were selected for lung histopathological evaluation. Rats were handled 24 h after LPS inhalation, taken out the lung tissues and washed with PBS to prepare sections. Hematoxylin Eosin (HE) staining: dewaxed and stained with hematoxylin for 5 min, returned to blue in 0.6% ammonia, rinsed and stained 3 min, dehydrated with ethanol, and sealed with neutral gum for observation and analysis. Isotype controls were processed without primary antibody incubation. Lung pathological changes was examined under a microscope (Eclipse 80i, Nikon, Japan) at 20x magnification. Immunohistochemical staining: Anti-Socs3 primary antibody (1:200, Abcam, UK) was added after the sections were deparaffinized and incubated at 37 °C for 1 h. Secondary antibodies and DAB staining were incubated according to the immunohistochemical staining kit (Maixin Biotechnology Co., Ltd., China) instructions. Then, lung sections were counterstained with hematoxylin. Staining was visualized under a microscope at 20x magnification. And the positive staining area was analyzed with Image J software.

### Pathology scores of lung tissues

According to previous articles [20, 21], 4 scores were used for lung histopathological scoring: 1) Alveolar hemorrhage and congestion; 2) Alveolar hemorrhage; 3) Neutrophil infiltration or aggregation in alveolar or vascular walls; 4) Alveolar wall thickening and/or transparent membrane formation. A 0–4 score was used according to lesion severity: (0 = no lesions or very mild lesions, 1 = mild lesions, 2 = moderate lesions, 3 = severe lesions, 4 = very severe lesions). The lung injury score was calculated as the sum of the scores of each index.

### Assessment of pulmonary edema levels

The lung tissues of the rats were collected, weighed and recorded immediately, and this weight was the wet weight. The lung tissue was then heated at 80 °C for forty-eight hours before being weighed and recorded as the dry weight of the rat lung tissue. The level of pulmonary edema was evaluated by calculating the ratio of wet weight to dry weight(W/D).

### Alveolar permeability measurement

After intratracheal instillation of LPS for 4 h or drug treatment for 24 h, 0.2 ml of 1% Evans blue staining solution was injected into caudal vein and killed 30 minutes later. Lung tissues were isolated and placed in 2 ml formamide solution for 48 h. Evans blue was collected from lung tissue and the absorbance was read at 620 nm. Rats performing Evans blue staining experiments were not used in other experiments.

### MPO activity measurement

Lung tissues from rats were collected and tissue homogenates were prepared in saline following the weight-to-volume ratios given according to the MPO assay kit (Nanjing Jiancheng, China). The supernatant was retained after centrifugation and the MPO activity in the lung tissue was measured.

### Cell counts in the BALF

A random small amount of BALF was put in the hematocrit plate (25 × 16) and remained for 3 minutes. The plate was centrifuged at 4 °C and 1500 r/min for 15 minutes, the obtained pellet was resuspended with PBS, and stained on glass slides with a drop of Wright-Giemsa stain solution for 15–30 min at room temperature. Cell countingwas performed under a microscope. The number of cells/mL = the total number of cells in 80 small squares/80 × 400 × 10,000 × dilution factor.

### Neutrophil culture

MEM medium and fetal bovine serum were prepared for culturing human neutrophils (Shanghai Chuntest Biotechnology Co. Ltd., Shanghai, China). Thaw the cryovial containing 1 ml of cell co-treatment. Take the pellet and add it to 2 ml of medium and mix well, place it in a petri dish containing medium, and then culture it in a cell incubator.

The cells of the 3rd-4th generation were divided into the control group (NC, LPS induction group (LPS), budesonide treatment group (LPS + BUD), N-acetylcysteine treatment group (LPS + NAC), mixed budesonide and N-acetylcysteine treatment group (LPS + co-treatment), miR-196b-5p mimic transfection group (LPS + co-treatment+miR-196b-5p mimic) and si-Socs3 transfection group (LPS + co-treatment+si-Socs3). Lipofectamine 2000 (miR-196b-5p mimic and si-Socs3 were purchased from Guangzhou Reebok Biological Co., Ltd.) was used to transfect miR-196b-5p mimic and si-Socs3 into cells, and 48 hours after transfection, 10 ng/mL LPS stimulated cells for 24 hours. BUD (2 ng/ml) and NAC (3 μg/ml) were given alone or in combination, while NC group and LPS group were treated with the identical amount of normal saline.

### Flow-through detection of CD35 and CD62 L surface expression

Neutrophils were prepared in single cell co-treatment, 10 μL each of anti-CD35(1:1000, Abcom, UK) and anti-CD62L(1:1000, Abcom, UK) antibodies labeled with fluorescein isothiocyanate (FITC) (Abeam, UK) were added, washed twice with PBS after incubated in the dark. PBS was then added to resuspend the cells, and the expression of CD35, CD62L and isotype controls were measured by flow cytometry (BD Biosciences, San Diego, CA, USA), and the fluorescence intensity was detected. Mean fluorescence intensity ratio = [fluorescence (test)-fluorescence (isotype control)] fluorescence (isotype control).

### Propidium iodide assay for NET synthesis

Quantify neutrophil extracellular trap network (NET) production by detecting the increase in fluorescence emission of propidium iodide (PI) in response to extracellular DNA binding. Add cells to 96-well plates containing assay buffer and incubate for 24 h at 37 °C before adding 10 mg/mL PI. Measure fluorescence levels at 360 nm and 612 nm using a fluorescence plate reader.

ReverseTranscription-PolymeraseChainReaction (RT–qPCR).

TRIzol RNA extraction kit (Thermo Fisher, US) is served by extracting the total RNA from the collected cells and tissues, and treated with RNase R at 37 °C, and then cDNA was reverse transcribed with the PrimeScript RT Master Mix (TaKaRa，Japan). miRNA and mRNA expression were detected using the SYBRGreen real-time PCR system. U6 and β-actin were deeded as internal references, and the primers were as follows: β-actin: forward: 5′-CCCAAGGCCAACCGCGAGAA-3′, reverse: 5′-CAGGAAGGAAGGCTGGAAGAG-3′. U6: forward: 5′-CGCTTCGGCAGCACATATAC-3′, reverse: 5′-AATATGGAACGCTCTA-3′. Interleukin (IL)-6: forward: 5′- CGGTCCAGTTGCCTTCTCCC-3′, reverse: 5′- AGGCTGGCATTTGTGGTTGG-3′. TNF-α: forward: 5′- AGGGCTCCAGGCGGTGCTTGTT-3′, reverse: 5′- ACGGCGATGCGGCTGATGGT-3′. IL-8: forward: 5′-CTTGGCAGCCTTCCTGATTT-3′, reverse: 5′- ACAACCCTCTGCACCCAGTT-3′. IL-10: forward: 5′-GCTCTGTTGCCTGGTCCTCC-3′, reverse: 5′-CTGCTCCACGGCCTTGCTCT-3′. miR-196b-5p: forward: 5′-ATCCTTCCTAGTCCAGCCTGAG-3′, reverse: 5′-ACCTGGCGGCACTCCTTA-3′. The PCR reaction conditions were as follows: pre-denaturation at 95 °C for 10 min, followed by 3 steps, denaturation at 95 °C for 15 s, and annealing at 60 °C for 30 s. There were 45 cycles in total. Finally, the experimental results are expressed as relative quantitative analysis of 2^−ΔΔCt^.

### Dual-luciferase reporter gene

The wild-type (WT) 3′-Untranslated Region (UTR) of Socs3-cDNA was synthesized by PCR and duplicated into a pmir reporter luciferase miRNA target expression vector to produce the WT-Socs3–3′-UTR. The mutants (MUT) of the Socs3–3′-UTR were based on the WT-Socs3–3′-UTR by mutating the nucleotides that could potentially bind to miR-196b-5p and inserting them into the pmir vector to generate the mutant vector MUT-Socs3–3′-UTR. These vectors and miR-196b-5p mimics or miR-negative controls (NC) were transiently transfected into 293 T cells using liposome 3000 reagent. Then,transfected 48 h later to detect the luciferase activity by utilizing a dual-luciferase reporter gene assay system.

### Western blotting assay

The total protein in each group was extracted and determined by BCA. 30 μg sample was taken for proceed to electrophoresis separation, transmembrane, placed in 5% TBST nonfat dry milk, and blocked for 1 h. Anti-Socs3 antibody (1:1000, Abcam, UK) diluted 1:2000 was used and incubated for 1 h, then the membrane was washed on a shaker. Secondary antibody (1:2000, Abcam, UK) was utilized and incubated for 1 h, then washed the membrane. Finally, the chemiluminescence reaction is carried out.

#### Enzyme-Linked Immunosorbnent Assay (ELISA)

IL-6, IL-8, IL-10, and TNF-α in rat BALF and neutrophil supernatants were quantified using ELISA kits (Thermo Fisher, US). Add the sample to the sample well, add biotin-labeled antibody, incubate at 37 °C for 2 h, then add horseradish peroxide-labeled streptavidin, and incubate for 1 h. Finally, the chromogenic solution was added for 30 min. Terminated the reaction, measured the OD value at 450 nm, and determined.

### Statistical analysis

Data are expressed as mean ± standard deviation. Comparisons between two groups were performed by t-test, and comparisons among multiple groups were performed by one-way ANOVA. Statistical analysis was performed using SPSS 26 software.

## Results

### Combined BUD and NAC treatment attenuates neutrophil recruitment to improve LPS-induced ALI

**Fig. 1:**
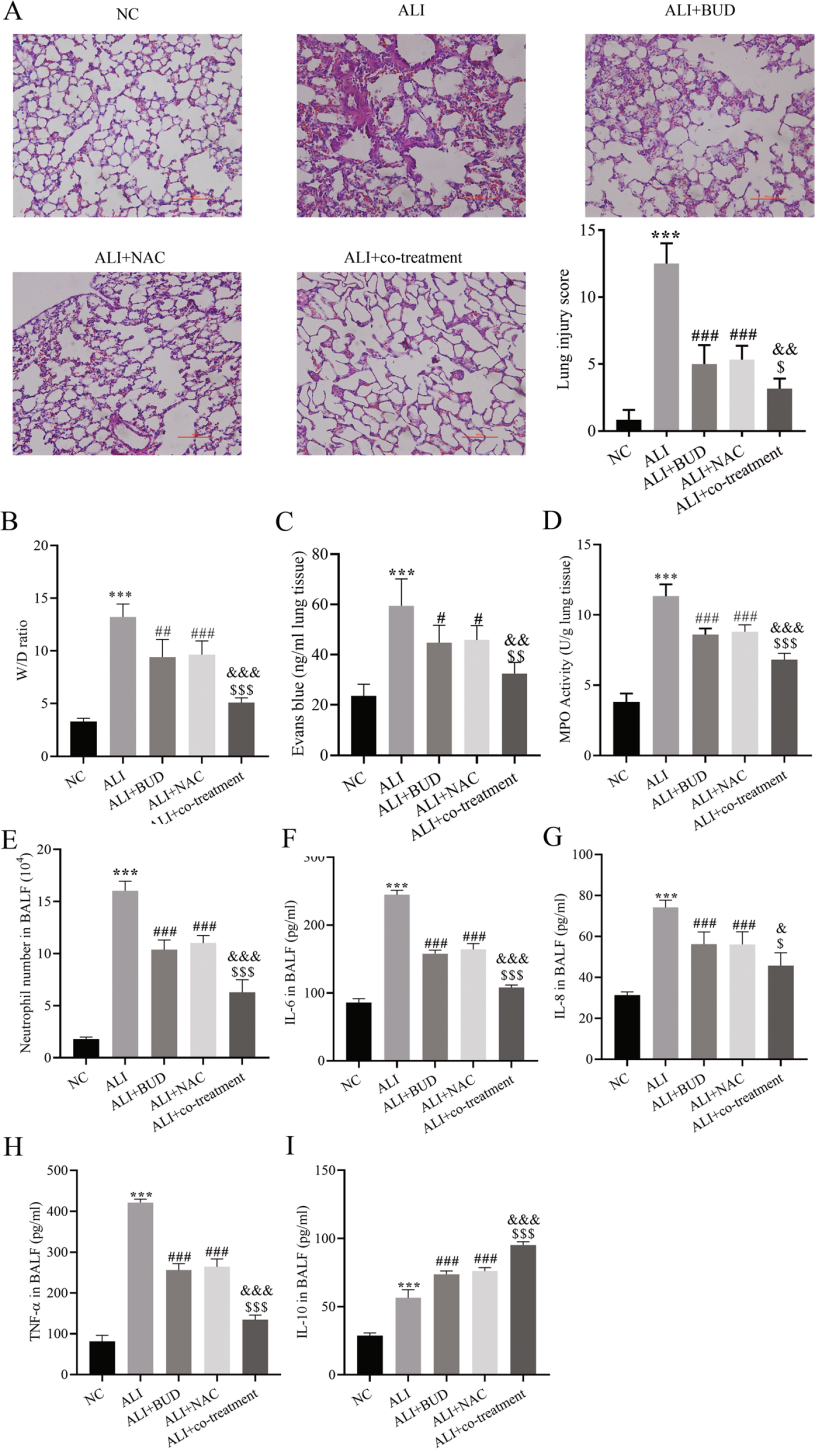
Combination therapy with BUD and NAC improves LPS-induced ALI. **A** Histopathological changes of lung injury and pathology scores of lung tissues (*n* = 6), **B** pulmonary edema level(*n* = 6), **C** Evans blue staining for alveolar permeability(*n* = 6), **D** MPO activity of lung tissue(*n* = 6), **E** neutrophil count in BALF(*n* = 6), **F**-**I** ELISA for cytokines in BALF(*n* = 6). compared with the NC group, ^**^ *P* < 0.01, ^***^ *P* < 0.001; compared with the ALI group, ^#^ *P* < 0.05, ^##^ *P* < 0.01, ^###^ *P* < 0.001; compared with the ALI + BUD group, ^$^ *P* < 0.05, ^$$^ *P* < 0.01, ^$$$^ *P* < 0.001; compared with the ALI + NAC group, ^&^ *P* < 0.05, ^&&^ *P* < 0.01, ^&&&^ *P* < 0.001

First, rats were treated with different concentrations of BUD or NAC, and lung injury scores were performed to select the best treatment concentration. The results showed that the optimal therapeutic concentrations of BUD and NAC were 0.36 mg / kg and 60 mg / kg, respectively (fig. S1). Further, the best concentration of BUD and NAC was used for inhalation treatment, the results of HE staining of rat lung tissue sections showed that LPS group had obvious lung injury. BUD and NAC alone or in combination could improve LPS induced lung injury, but the effect of combined treatment was better than that of each drug alone (Fig. 1A).

Further, wet/dry weight ratio of lungs and Evans blue staining are used to judge alveolar permeability and pulmonary edema, respectively, and the results showed that the alveolar permeability of the model group increased, and the pulmonary edema was significantly aggravated. Treated with BUD and NAC alone or the combination therapy significantly reduced pulmonary edema and alveolar permeability in rats, while the combination therapy group significantly decreased pulmonary edema and alveolar permeability compared with the monotherapy group (Fig. 1B, C).

The increase in MPO activity reflects the accumulation of neutrophils in the lung [22], examined lung MPO activity and found that both BUD and NAC treatment groups alone or in combination significantly reduced lung MPO activity in rats. Nevertheless, the treatment group showed the opposite symptoms, the MPO activity was obviously decreased in the combined treatment group (Fig. 1D).

Furthermore, we measured neutrophil counts in the BALF of rats, and both BUD and NAC treatment alone or in combination significantly decreased BALF neutrophil counts in ALI rats, and the neutrophil counts in the combination treatment group were less than those in the monotherapy group (Fig. 1E), indicating that combined BUD and NAC treatment reduced neutrophil recruitment compared with individual treatments.

The cytokines in BALF were detected by ELISA. It was found that the pro-inflammatory cytokines IL-6, IL-8 and TNF-α in BALF in LPS group And the expression level of anti-inflammatory cytokine IL-10 in NC group was significantly higher than that in NC group. The level of IL-10 was higher in the LPS group compared with the NC group, which may be due to the significant raise the levels of TNF-α and IL-10 after LPS-induced upregulation [23]. BUD and NAC treatment alone or in combination significantly inhibited the production of IL-6, IL-8, and TNF-α and further promoted IL-10 production, and the combination treatment was significantly more effective than the individual treatments (Fig. 1F-I).

The above results indicated that the combined treatment with BUD and NAC was more effective in improving LPS-induced ALI in rats by attenuating the recruitment of neutrophils and inhibiting the inflammatory response than either treatment alone.

### Combined BUD and NAC treatment improves LPS-induced neutrophil activation

**Fig. 2:**
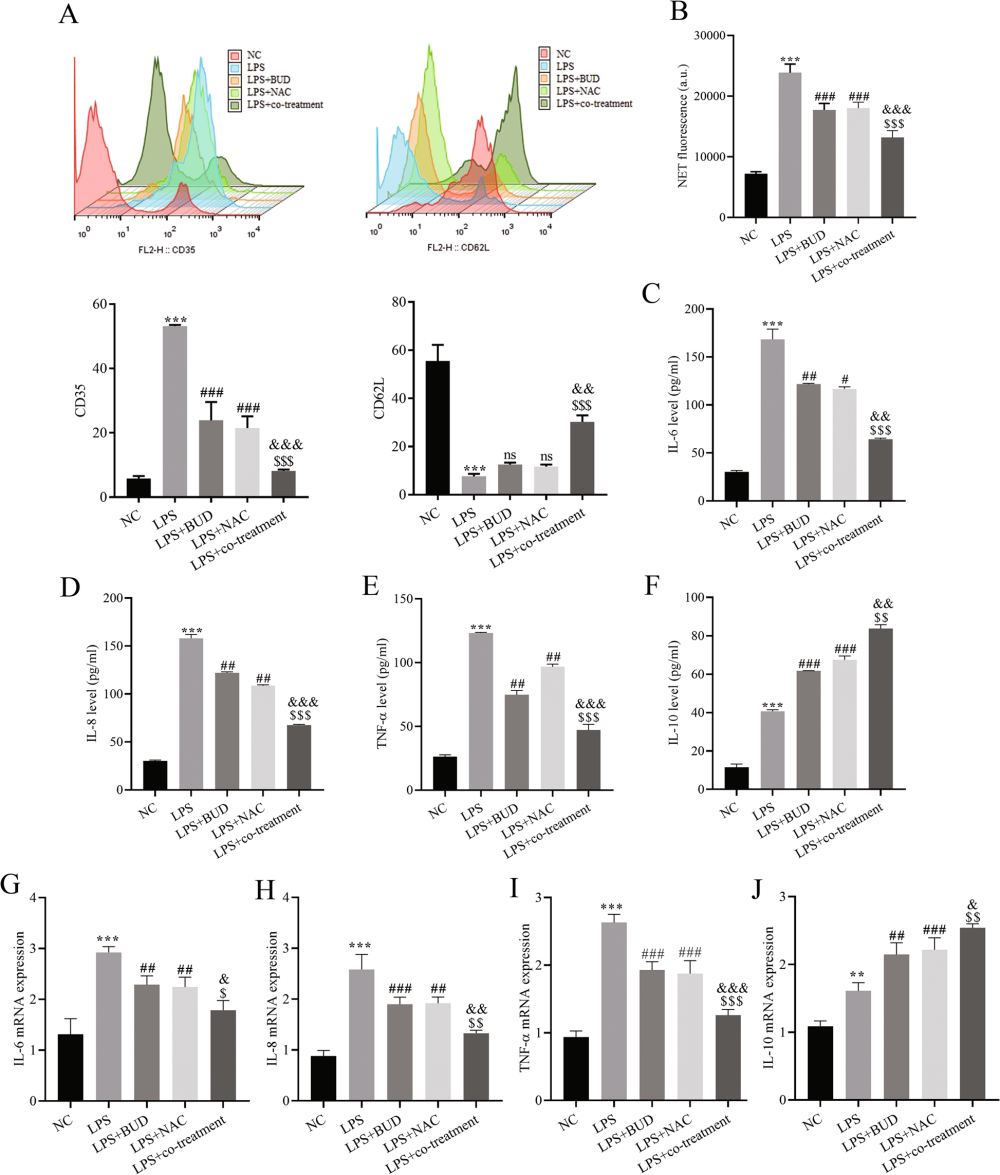
Combination therapy with BUD and NAC improves the neutrophil-induced inflammatory response. **A** Flow cytometry for surface expression of CD35 and CD62 L; **B** Neutrophil extracellular trap network formation assay; **C**-**F** ELISA for cytokine expression; G-J. RT–qPCR for cytokine mRNA expression. Compared with the NC group, ** *P* < 0.01, *** *P* < 0.001; compared with the LPS group, # *P* < 0.05, ## *P* < 0.01, ### *P* < 0.001; compared with the LPS + BUD group, $ *P* < 0.05, $$ *P* < 0.01, $$$ *P* < 0.001; compared with the LPS + NAC group, & *P* < 0.05, && *P* < 0.01, &&& *P* < 0.001

The expression levels of CD35 and CD62L on the surface of neutrophils were detected by flow cytometry. LPS induced treatment significantly increased the expression of CD35 and decreased the expression of CD62L. BUD and NAC alone reduced the expression of CD35, and the effect of the combined treatment group was more significant than that of any single treatment group (Fig. 2A). BUD and NAC alone have an up-regulation effect on CD62L, but there is no significant difference. The combined treatment group can significantly increase the expression of CD62L (Fig. 2A). The analysis results of propidium iodide show that LPS treatment significantly promoted the generation of NETs, while BUD and NAC alone or combined inhibited the generation of NETs, and the inhibitory effect of the combined treatment group was more obvious that of the single treatment group (Fig. 2B). This further confirmed that combined treatment with BUD and NAC was more effective in inhibiting the activation of neutrophils than the individual treatments.

The cell culture supernatant was collected. ELISA detection is an efficient method to detect the levels of IL-6, IL-8, TNF-α and IL-10. The data showed that the above pro-inflammatory factors IL-6, IL-8, TNF-α were prominently increased in the model group and decreased in each treatment group, especially in the combined treatment group of BUD and NAC. While the anti-inflammatory cytokine IL-10 in the cell supernatant of the LPS group were significantly increased and treatment group promoted IL-10 production (Fig. 2C-F).

RT–qPCR assays revealed that the LPS group upregulated IL-6, IL-8, TNF-α and IL-10 mRNA expression. BUD and NAC treatment alone or in combination significantly downregulated the expression levels of IL-6, IL-8, and TNF-α mRNA and further upregulated levels of IL-10 mRNA, and the effect of the combination treatment was better than that of either individual treatment (Fig. 2G-J). In brief, the activation of neutrophils promoted an inflammatory response, while the combined treatment of BUD and NAC effectively suppressed the neutrophil-induced inflammatory response.

### Combined BUD and NAC treatment improves the neutrophil-induced inflammatory response by suppressing miR-196b-5p expression

**Fig. 3:**
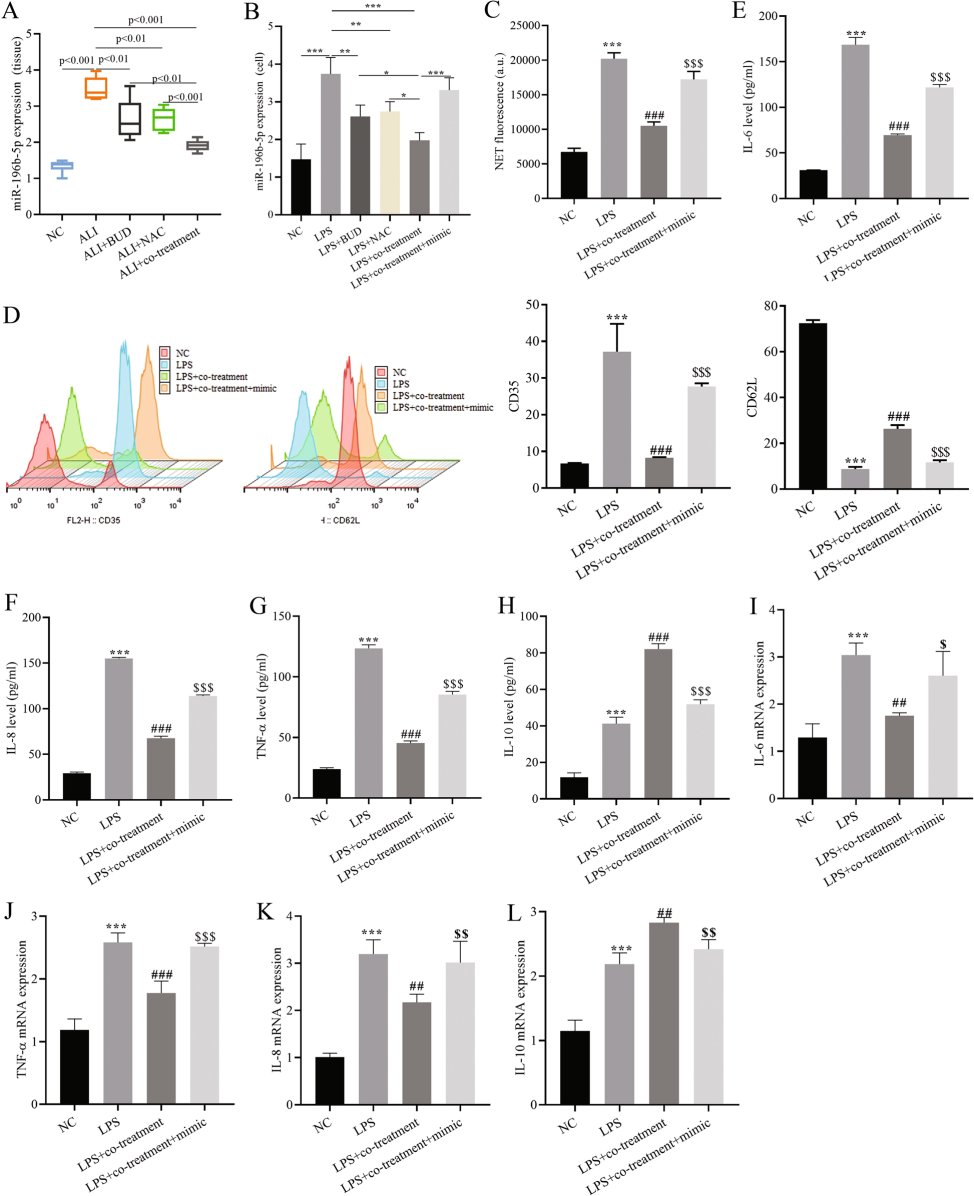
Effect of miR-196b-5p expression on the neutrophil-induced inflammatory response. **A** Expression of miR-196b-5p in lung tissue; **B** Expression of miR-196b-5p in cells; **C** Neutrophil extracellular trap network formation assay; **D** Flow cytometry for CD35 and CD62 L surface expression; E-H. ELISA for cytokine expression; **G-J** Expression of cytokine mRNA. compared with the NC group, ** *P* < 0.01, *** *P* < 0.001; compared with the LPS group, # *P* < 0.05, ## *P* < 0.01, ### *P* < 0.001; compared with the LPS + co-treatment group, $ *P* < 0.05, $$ *P* < 0.01, $$$ *P* < 0.001. mimic represents mir-196b-5p mimic

RT–qPCR assays revealed that the LPS group up-regulated miR-196b-5p expression in in tissues and cells (Fig. 3A and B). The data indicated that the expression levels of miR-196b-5p in LPS + BUD group, LPS + NAC group and LPS + co-treatment group were highly down-regulated compared with LPS group, and the down-regulation level in LPS + co-treatment group was remarkably lower than that in LPS + co-treatment group LPS + BUD and LPS + NAC group. Transfection of miR-196b-5p mimic restored the inhibitory effect of BUD and NAC on miR-196b-5p expression (Fig. 3B), demonstrating that the combined treatment with BUD and NAC could effectively inhibit miR-196b-5p expression. The analysis results of propidium iodide reveal that LPS treatment extremely promoted the generation of NETs, while the combined BUD and NAC treatment inhibited the generation of NETs, and transfection of the miR-196b-5p mimic restored the inhibitory effect of combined BUD and NAC treatment on the generation of NETs (Fig. 3C).

The surface expression of CD35 and CD62L were assessed by flow cytometry, and the above results indicated that LPS group regulated the expression of CD35 and CD62L in neutrophils significantly lower and higher than NC group. The combined treatment with BUD and NAC reduced CD35 expression and increased CD62 L expression, while transfection with miR-196b-5p mimic showed the opposite symptoms (Fig. 3D). The above results demonstrated that the combined BUD and NAC treatment inhibited neutrophil activation by suppressing the expression of miR-196b-5p.

ELISA data showed that the levels of the inflammatory factors including IL-6, IL-8, and TNF-α in the neutrophil culture supernatant of the LPS+ co-treatment group were remarkably lower than those of the LPS group, and increased the anti-inflammatory cytokine IL-10, while transfection with miR-196b-5p mimic reversed the inhibitory effect of the combined BUD and NAC treatment on IL-6, IL-8, and TNF-α and their promoting effect on IL-10 (Fig. 3E-H).

RT–qPCR assays were applied to assess the content of IL-6, IL-8, and TNF-α in the cells of the LPS + co-treatment group. The mRNA expression levels of IL-8 and TNF-α in the LPS + co-treatment group were lower than those in the LPS group, while the expression of IL-6, IL-8 and TNF-α after transfection of miR-196b-5p simulant The mRNA expression level increased significantly, and the expression level of IL-10 was significantly lower than that in LPS + co-treatment group (Fig. 3I-L).

### miR-196b-5p targets the downregulation of Socs3 expression

**Fig. 4:**
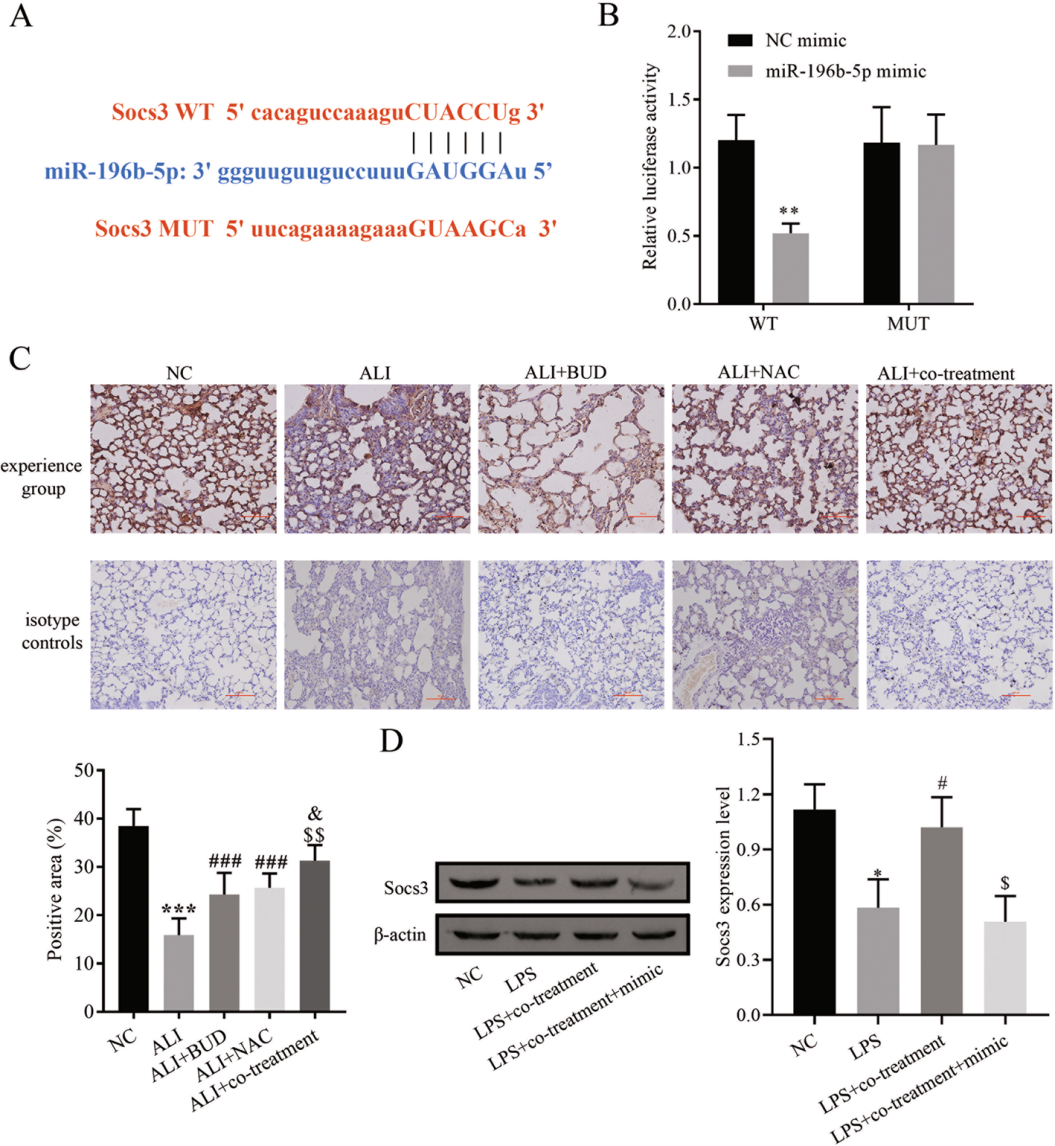
miR-196b-5p targeted downregulation of Socs3. **A** Binding sites of miR-196b-5p and Socs3; **B** Dual luciferase reporter gene; **C** Immunohistochemical staining for Socs3 expression; **D** Western blot for Socs3 expression. Compared with the NC group, ** *P* < 0.01, *** *P* < 0.001; compared with the LPS group, # *P* < 0.05; compared with the LPS + co-treatment group, $ *P* < 0.05. mimic represents mir-196b-5p mimic

The existence of binding sites for miR-196b-5p and Socs3 was predicted by the StarBase website (Fig. 4A). In order to test the targeting relationship between miR-196b-5p and Socs3, the wild-type Socs3 luciferase promoter plasmid (Socs3 3′UTR-WT) and mutant Socs3 luciferase promoter plasmid (Socs3 3′UTR-MUT), and dual luciferase reporter genes were assayed for luciferase activity. Transfection with miR-196b-5p mimic reduced the luciferase activity of Socs3 3′UTR-WT but had no effects in the luciferase activity of Socs3 3′UTR-MUT (Fig. 4B).

The results of Immunohistochemical detection of Socs3 expression in lung tissues revealed that the LPS group dramatically reduced expression, while the expression of Socs3 in the combined treatment group of BUD and NAC was significantly decreased and higher than that of the single treatment group (Fig. 4C). Western blotting was applied to assess the expression level of Socs3 in neutrophils, and the results found that the expression of Socs3 in the LPS group was apparently higher than NC group, while the combined treatment with BUD and NAC promoted the expression of Socs3, and transfection of miR-196b-5p mimic reversed this effect on the expression of Socs3 (Fig. 4D), further confirming that miR-196b-5p caused downregulation of Socs3 expression.

### Combined BUD and NAC Treatment Attenuates the Neutrophil-Induced Inflammatory Response via the miR-196b-5p/Socs3 Molecular Axis

**Fig. 5:**
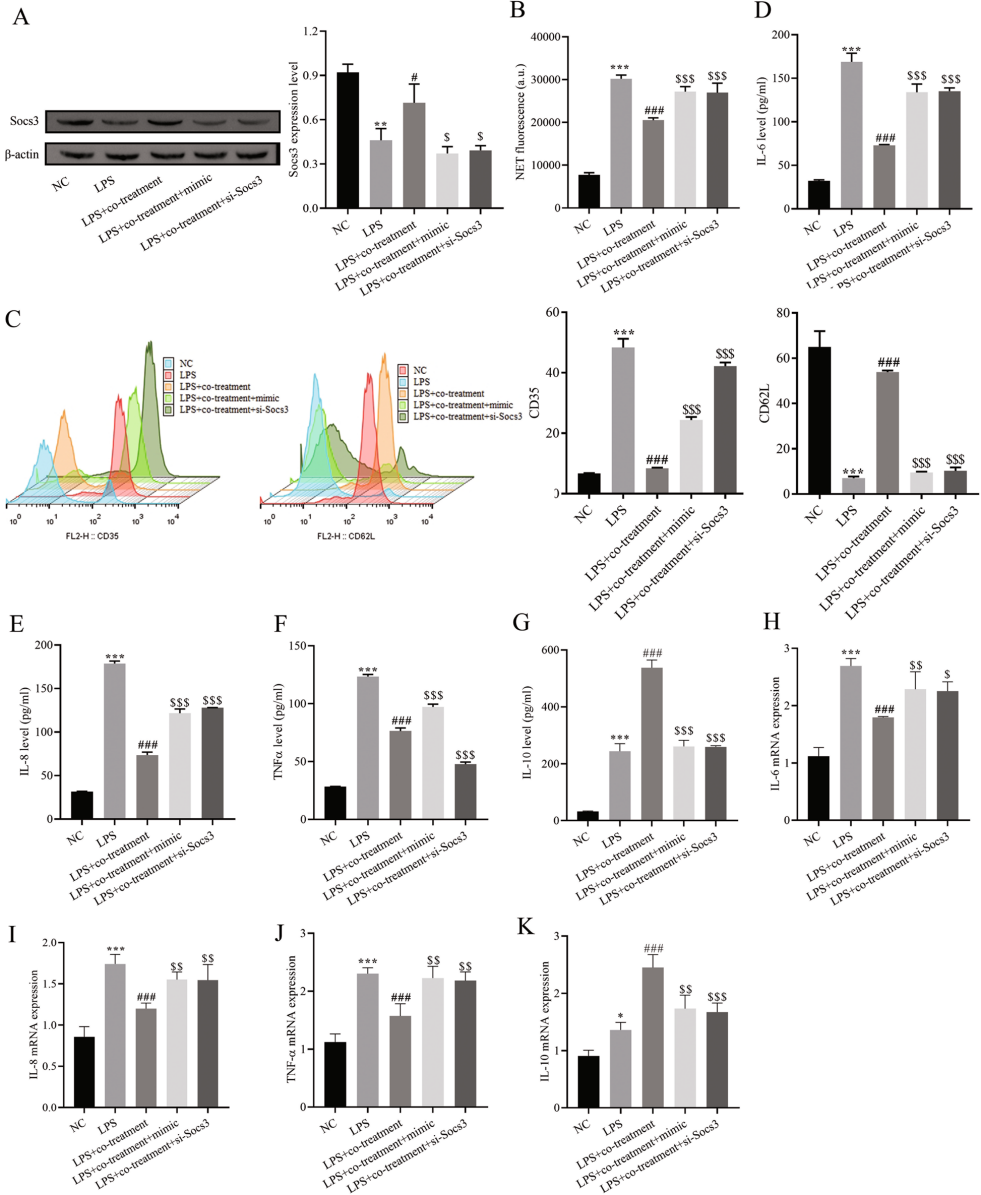
Attenuation of the neutrophil-induced inflammatory response by the miR-196b-5p/Socs3 molecular axis. **A** Western blot for Socs3 expression; **B** Neutrophil extracellular trap network formation assay; **C** Flow cytometry for CD35 and CD62 L surface expression; **D**-**G** ELISA for cytokine expression; H-K. RT–qPCR for cytokine mRNA expression. Compared with the NC group, *** *P* < 0.001; compared with the LPS group, # *P* < 0.05, ## *P* < 0.01, ### *P* < 0.001; compared with the LPS + co-treatment group, $ *P* < 0.05, $$ *P* < 0.01, $$$ *P* < 0.001. mimic represents miR-196b-5p mimic

Western blotting, flow cytometry, ELISA and RT–qPCR, and PI staining were used to assess the expression levels of Socs3, CD35, CD62 L and inflammatory factors and the production of NETs, and the results confirmed that the combined treatment of BUD and NAC upregulated the expression levels of Socs3, CD62 L and the factor IL-10, down-regulated the expression of CD35 and IL-6, IL-8, and TNF-α, and inhibited the production of NETs, compared with the LPS group (Fig. 5A-K). Overexpression of miR-196b-5p and knockdown of Socs3 selectivity reversed the effects of combined treatment with BUD and NAC on the promotion of Socs3, CD62 L and anti-inflammatory factors and the inhibition of CD35, pro-inflammatory factors and NETs (Fig. 5A-K).

## Discussion

ALI is associated with high incidence rate and mortality in the world, and its pathogenesis is complex and treatment methods are limited, which is still the main challenge faced by clinicians [24, 25].

Studies have shown that enlarged capillary permeability, interstitial and alveolar edema, release of inflammatory mediators, and neutrophil infiltration [26]. Neutrophil infiltration and accumulation is one of the most important pathological features of ALI [27]. It has been reported that neutrophils receive signals from the apoptotic program when the body is infected, inducing the release of NETs, which attract and eradicate bacteria [28]. Therefore, NET is an important indicator to detect neutrophil activation. MPO activity is a vital indicator of neutrophil migration to the lung. After stimulated by LPS, abundant polymorphonuclear nuclei collected from peripheral blood migrate into the lung, resulting in counts of MPO production, ultimately lead to serious inflammatory reaction and injury the lung tissue [29]. In addition, the migration of neutrophils from the circulation to inflammatory areas involves the regulated expression of adhesion molecules on the leukocyte surface. CD62 L has great effects in the initial attachment of leukocytes to endothelial cells and enables rapid shedding of neutrophils by proteolytic lysis after activation [30]. A previous study found that functional blockade of CD62 L resulted in neutrophil recruitment to the inflamed lung [31]. Evidence of neutrophil activation with increased expression of CD35 was found in severe asthma patients [32]. In addition, it was confirmed in the LPS induced ALI model that it can induce the release of a series of inflammatory factors such as interleukin and tumor necrosis factor [33], which is a key factor in inducing lung inflammation. Among them, IL-6 is a pleiotropic cytokine that induces endothelial permeability and cell recruitment, as well as B cell maturation and T cell survival to exert pro-inflammatory effects, and can also downregulate the synthesis of IL-1 and TNF to exert anti-inflammatory effects [34, 35]. In accordance with the literature, we found that in LPS induced ALI, IL-6 usually acts as a proinflammatory cytokine, for example, Li [27], Ju [36] and Wang [37] all found that LPS significantly induced IL-6 expression in the BALF of ALI mice. Our results showed that LPS increased MPO activity, net release, CD35 expression and decreased CD62L expression in lung tissue. In addition, LPS also promoted the production of pro-inflammatory cytokines IL-6, IL-8, TNF-α and the level of the anti-inflammatory factor IL-10 (this may be due to LPS induced TNF-α After upregulation, IL-10, as a counter regulatory cytokine, will significantly increase its level). This indicates that LPS can induce neutrophil activation and aggregation, which in turn induces lung inflammation.

BUD and NAC are often used in the treatment of bronchopneumonia [38], and studies have shown that BUD and NAC can alleviate the lung injury rats which are LPS-induced by repressing the inflammatory response [9, 39, 40]. However, the effect of combined treatment with BUD and NAC has not been reported thus far. We used LPS to establish ALI rats and cell models, and the results confirmed that BUD and NAC treatment alone or in combination significantly improved LPS-induced induced increase in alveolar permeability, pulmonary edema, reduce neutrophil activation and aggregation, and reduce pulmonary inflammatory response. The effect of combined therapy is better than that of single therapy.

miRNAs are non-coding small RNAs that adjust the posttranscriptional genes by binding to the 3′ untranslated regions (UTRs) of target mRNAs, and then inhibit translation and degrade mRNA [41]. Studies have shown that miR-196b-5p has a vital function in gene regulation in the lung and other respiratory and digestive tract tissues [42]. In addition, miR-196b-5p was confirmed to target and regulate Socs3 expression [18]. Socs3 is a key suppressor of granulopoiesis, and many researches have also reported the function of Socs3 in inflammatory response and lung injury, such as the anti-inflammatory effects driven by the binding of Socs3 to microtubule plus-binding protein to protect lung endothelial function [43]. Socs3 promotes the repair of rat lung injury by inhibiting JAK2/STAT3 pathway activation and thereby inhibiting inflammatory factors [44], and residents alveolar macrophages inhibit airway allergic reaction through the constitutive secretion of Socs3 in extracellular vesicles [45]. In addition, Socs3 was also confirmed to inhibit the release of inflammatory factors in neutrophils [46]. For example, in LPS-induced ALI, Socs3 deficiency was shown to disrupt the balance between pro-inflammatory cytokines (TNF-α and IL-1β) that promote tissue destruction and anti-inflammatory cytokines (IL-10 and IL-13) that promote disease resolution, accelerating LPS-induced lung inflammation and injury [47]. Our study found that the expression of Socs3 was decreased in LPS induced ALI rats and cell models, and BUD and NAC inhibited the inflammatory factors IL-6, IL-8 and TNF-α and promote the production of IL-10 by inhibiting the targeted downregulation of Socs3 by miR-196b-5p, and alleviate LPS induced inflammatory response and lung injury.

The above results suggest that the ability of the combination treatment of BUD and NAC alleviated LPS induced ALI in rats by suppressing the target down-regulation of Socs3 by miR-196b-5p. These findings elucidate the mechanism by which combined BUD and NAC therapy ameliorates LPS-induced ALI, suggesting that miR-196b-5p may be a potential therapeutic target for ALI.

## Conclusions

In conclusion, we found that the combination of BUD and NAC improved LPS-induced ALI by inhibiting pulmonary neutrophil recruitment better than treatment alone. Its molecular mechanism is to inhibit the expression of miR-196b-5p, up-regulate the expression of Socs3, and then inhibit the recruitment of neutrophils in the lungs.

## Supplementary Information

**Additional file 1.**

**Additional file 2.**

**Additional file 3.**

## Data Availability

The authors confirm that the data supporting the findings of this study are available within the article and/or its supplementary material.

## Abbreviations

ALI: Acute lung injury
BUD: Budesonide
NAC: N-acetylcysteine
LPS: Lipopolysaccharide
BALF: Bronchoalveolar lavage fluid
Socs3: Suppressor of cytokine signaling pathway 3
FITC: Fluorescein isothiocyanate
NET: Neutrophil extracellular trap network
PI: Propidium iodide
W/D: Wet/dry weight
UTRs: Untranslated regions
