# Supplementary figures and images for: Combination therapy with budesonide and N-acetylcysteine ameliorates LPS-induced ALI by attenuating neutrophil recruitment through the miR-196b-5p/Socs3 molecular axis
Source: BMC Pulm Med. 2022 Oct 26;22:388. doi: 10.1186/s12890-022-02185-7 (PMC9608916; doi:10.1186/s12890-022-02185-7)

full-length gels and blots:

1. 4D

Socs3


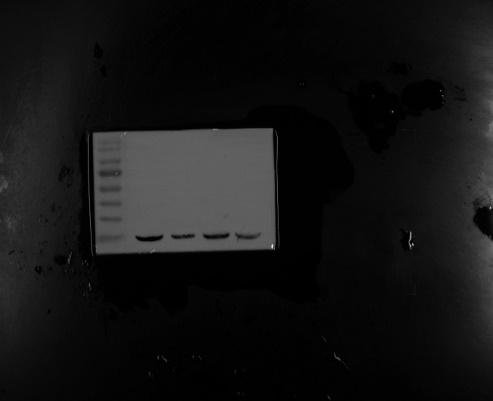


β-actin


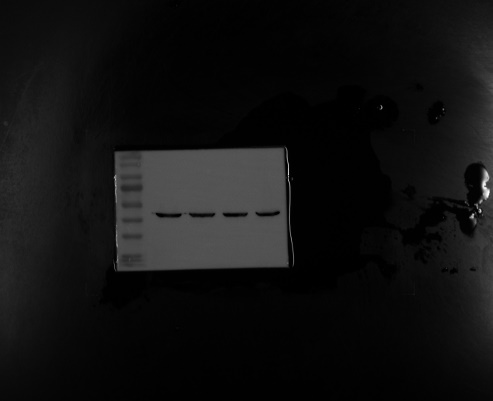


5A

1. Socs3


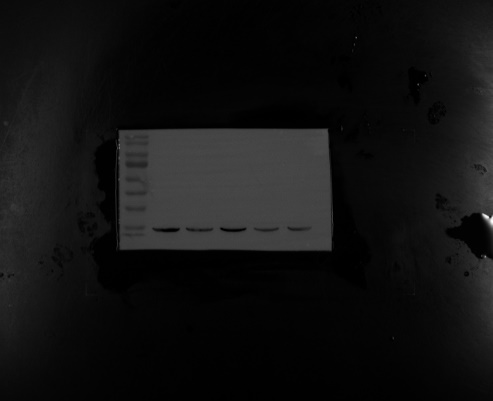


2. β-actin


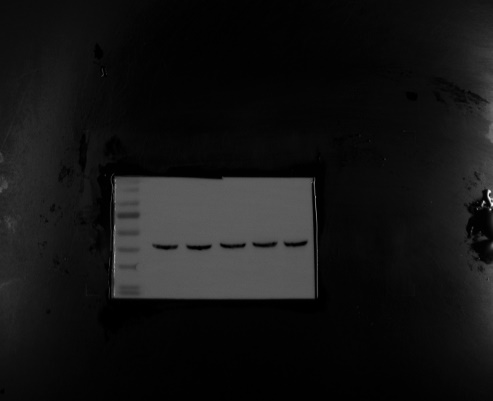

Supplement: Supplementary file 1 — Additional file 1. [file 12890_2022_2185_MOESM1_ESM.docx]
